# Evolutionary history and spatio-temporal dynamics of dengue virus serotypes in an endemic region of Colombia

**DOI:** 10.1371/journal.pone.0203090

**Published:** 2018-08-29

**Authors:** Cinthy L. Jiménez-Silva, María Fernanda Carreño, Ayda Susana Ortiz-Baez, Luz Aida Rey, Christian Julián Villabona-Arenas, Raquel E. Ocazionez

**Affiliations:** 1 Laboratorio de Arbovirus, Centro de Investigaciones en Enfermedades Tropicales (CINTROP), Universidad Industrial de Santander, Bucaramanga, Colombia; 2 Unidad de Epidemiología Clínica, Escuela de Medicina, Universidad Industrial de Santander, Bucaramanga, Colombia; 3 Laboratoire TransVIHMI, Institut de Recherche pour le Développement (IRD), Université de Montpellier, Montpellier, France; 4 Laboratoire d'Informatique, de Robotique et de Microélectronique de Montpellier (LIRMM), Institut de Biologie Computationnelle (IBC), Université de Montpellier, Montpellier, France; Instituto Nacional de Salud Pública, MEXICO

## Abstract

Dengue is a prevalent disease in Colombia and all dengue virus serotypes (DENV-1 to -4) co-circulate in the country since 2001. However, the relative impact of gene flow and local diversification on epidemic dynamics is unknown due to heterogeneous sampling and lack of sufficient genetic data. The region of Santander is one of the areas with the highest incidence of dengue in Colombia. To provide a better understanding of the epidemiology of dengue, we inferred DENV population dynamics using samples collected between 1998 and 2015. We used Bayesian phylogenetic analysis and included 143 new envelope gene sequences from Colombia, mainly from the region of Santander, and 235 published sequences from representative countries in the Americas. We documented one single genotype for each serotype but multiple introductions. Whereas the majority of DENV-1, DENV-2, and DENV-4 strains fell into one single lineage, DENV-3 strains fell into two distinct lineages that co-circulated. The inferred times to the most recent common ancestors for the most recent clades of DENV-1, DENV-2, and DENV-4 fell between 1977 and 1987, and for DENV-3 was around 1995. Demographic reconstructions suggested a gradual increase in viral diversity over time. A phylogeographical analysis underscored that Colombia mainly receives viral lineages and a significant diffusion route between Colombia and Venezuela. Our findings contribute to a better understanding of the viral diversity and dengue epidemiology in Colombia.

## Introduction

Dengue disease is highly prevalent in tropical countries due to climate, population growth, unplanned rapid urbanization and increased travel and trade [1,2]. Consequently, the global burden of dengue disease is high: a total of 58.4 million (23.6 million–121.9 million) apparent cases in 2013 and 1.14 million (0.73 million–1.98 million) disability-adjusted life-years [3]. Dengue is caused by four closely related viruses referred to as serotypes (DENV-1, DENV-2, DENV-3, and DENV-4), which are further subdivided into genotypes [4]. A higher risk of severe dengue is attributed to the co-circulation of multiple serotypes due to antibody-dependent enhancement of infection [2] and to some particular strains [5]. Therefore, documenting serotype prevalence and the dynamics of genetic variants help forecast epidemic impact, ensuing epidemic management and preparedness.

Colombia is a tropical country in the northwest of South America with high dengue incidence. For instance, a total of 752,429 cases were reported during 1980–2007 [6–9]: the country-wide 2001/2002, 2010, and 2013/2014 epidemics had 143,820, 147,257, and 247,075 notified dengue cases, respectively. DENV-2, DENV-1, and DENV-4 were reported for the first time in the country in 1971, 1977, and 1982, respectively [10]. DENV-3 was reported only once in 1975 and re-emerged again in 2001 [10,11]; the four DENV serotypes have been frequently documented in Colombia from that year onwards [9,11,12].

A total of six Colombian states (out of 32) accumulate more than half of the cases in the country: Valle del Cauca (19%), Santander (12%), Antioquia (8%), Norte de Santander (7%), Huila (6%), and Tolima (6%) [7,9,12]. The region of Santander is located in the northeast of Colombia and comprises the states of Santander and Norte de Santander. Previous studies reported changes in the predominance of serotypes over time in this region [11,13–15]. The argument over whether there is a genetic basis or there are random fluctuations that explain the temporal distribution of serotypes has not been settled yet.

Previous studies [16–19] that focused on the molecular epidemiology of dengue in Colombia included sequence data collected up to 2008 and did not include DENV-4. Consequently, many aspects of the local epidemiology remain unclear. In the present study, we obtained 143 new envelope gene sequences from serum samples collected in the region of Santander and six sequences from serum samples collected in other regions between 1998 and 2015. We provide insights into the evolution and population dynamics of all four dengue serotypes in the region of Santander, which help understanding dengue epidemiology in Colombia and might be relevant for future control programs, including vaccination.

## Materials and methods

### Study sites

**Fig 1:**
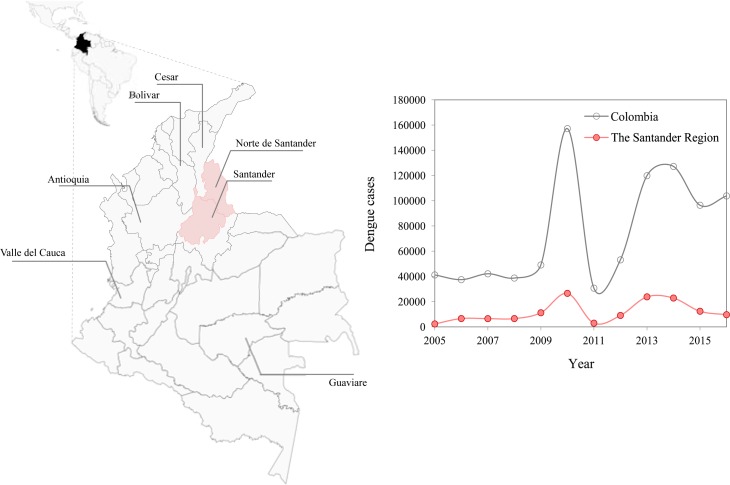
Geographical origin of the samples from Colombia and dengue incidence. The region of Santander (Santander and Norte de Santander states) is colored in red.

Serum samples were collected from the metropolitan area of Bucaramanga in the state of Santander and the province of Ocaña in the state of Norte de Santander; these constitute the region of Santander that is located in the central northern part of Colombia [Fig 1]. Bucaramanga is the capital city of Santander and together with three nearby municipalities constitutes the seventh largest metropolitan area of the country (around 1.4 million inhabitants). It has an average altitude of ~1,200 meters above the sea, an annual mean temperature of 25°C and an average amount of annual precipitation of 1,041 mm. The city’s urban mass transportation system moves over 100,000 passengers daily and extends to the Metropolitan area [20]. Moreover, Bucaramanga is a transportation hub for the northeast of Colombia and it has a bus terminal where 1.4 million passengers commute to diverse regions of the country on a yearly basis [21]. Bucaramanga’s airport has national and international traffic operations, which mobilized more than 1.2 million people in 2010. At least 94% of dengue cases from Santander were reported in the metropolitan area of Bucaramanga [7,9], and it was one of the participant cities in the dengue vaccine CYD-TDV clinical trial [22].

The province of Ocaña is a conurbation of 10 municipalities in an area of 2,065 meters above the sea in the state of Norte de Santander. Ocaña is its capital and its third largest municipality (around 98,229 inhabitants in 2014). This city has an annual mean temperature of 22°C and an average amount of annual precipitation of 1,000 mm. Cucuta is the capital city of Norte de Santander, it is bordered by Venezuela which makes it an important commercial city. Ocaña is located at a distance of 197 km (122 mi) from Cucuta. Ocaña has a small airport with only regular flights to Cucuta [23].

### Viral strains

DENV strains were obtained from the collection of the Laboratorio de Arbovirus, Centro de Investigaciones en Enfermedades Tropicales (CINTROP), Universidad Industrial de Santander, Bucaramanga. Viruses were isolated by culturing in C6/36 mosquito cells from patient sera collected in previous studies [11,14,15,24,25]. Sera were collected either for routine dengue laboratory diagnosis at medical institutions—for which an Institutional Ethics Committee approval was not required—or collected from patients enrolled in cross-sectional clinical trials, which were approved by the Research Ethics Committee of the Universidad Industrial de Santander. In the latter, an informed consent from each patient was obtained. All virus samples were analyzed anonymously. We selected samples to represent years in which each virus serotype was recorded between 1998 and 2015: 137 samples from the region of Santander (60 DENV-1, 33 DENV-2, 39 DENV-3, and 11 DENV-4) and six samples that were available from the Valle del Cauca, Bolivar, and Cesar states (southwestern and northeastern regions).

### Full-length envelope gene (E-gene) sequencing

Viral RNA was extracted directly from supernatants of DENV-infected C6/36 cells using the QIAamp Viral RNA mini kit (Qiagen). The RNA was transcribed to cDNA using RevertAid reverse transcriptase (Thermo Scientific, USA) and random hexamer primers (Thermo Scientific). The full E-gene (~1485 pb) was amplified by PCR using DENV serotype specific primers [S1 Table] and Thermo Scientific Taq DNA Polymerase according to manufacturer instructions. Each reaction of DNA amplification produced overlapping amplicons of 863–1520 nucleotides in length. Amplicons were sequenced using a Sanger sequencing commercial service (Macrogen DNA Sequencing Service, Seoul Korea). Sequence assembly was performed with CLC Genomics Workbench 4.5 (CLC Bio, Denmark) and sequences were deposited in GenBank [S2 Table].

### Data selection

Available full-length E-gene accessions with known location and sampling date were retrieved from GenBank (last accessed in March 2017). The total dataset (n = 9.669) was aggregated by serotype (3676 of DENV-1, 2925 of DENV-2, 1746 of DENV-3 and 1322 of DENV-4) and analyzed separately. Identical sequences from the same country and year were removed using the UCLUST algorithm in the USEARCH v.10.0.240 software [26]. The filtered datasets (3452 of DENV-1, 2789 of DENV-2, 1645 of DENV-3 and 1100 of DENV 4 Sequences) were combined with novel Colombian isolates, aligned using MUSCLE v.3.8 [27] and visualized using Bioedit v.7.2.5 software [28]. Preliminary phylogenetic analyses were done using Maximum Likelihood (ML) methods with a non-parametric bootstrap using PhyML v.3.1 software [29] in order to identify genotypes and to focus the analysis in the genotypes that were observed in Colombia [S2 Fig].

Sequences from the same year were downsampled when they fell within a monophyletic group by country and were overrepresented. The resulting datasets (DENV-1 = 118, DENV-2 = 118, DENV-3 = 66 and DENV-4 = 77) had only sequences from the region of the Americas because the phylogenetic trees showed a single introduction of each genotype into this region from non-American countries.

A Likelihood Mapping Analysis in TREE-PUZZLE v.5.3 [30] software and a substitution saturation analysis in DAMBE v.6 [31] showed that there was enough phylogenetic information in each dataset [S1 Fig]. All datasets were free of recombination signal following a pairwise homoplastic index test using SplitsTree v.4.12.6 [32] (pairwise homoplastic index ≤ 0.5 for each dataset).

### Data analysis

Haplotype diversity and nucleotide diversity were calculated using the software DNAsp v.5 [33]. For each dataset, the best-fit model of nucleotide substitution was selected based on Akaike Information Criterion (AIC) using the APE package v.3.4.2 [34] and the R v.3.3.0 software [35]; The Tamura and Nei plus invariant and discrete gamma models were the best-fit nucleotide substitution models for DENV-1 and -2, and the generalized time reversible plus invariant and discrete gamma models were the best-fit nucleotide substitution models for DENV-3 and -4. Regression of root-to-tip genetic distance against sampling time [S3 Table] using TempEst v 1.5 [36], showed that there was sufficient temporal signal in each dataset to proceed with phylogenetic molecular clock analyses. The best-fit clock model (strict vs. relaxed) and the best-fit demographic model (among constant size, exponential growth, *skyride* and *skygrid* models), were selected via path sampling (PS) and stepping-stone (SS) methods (100 steps with 5 million iterations each) [37]. In all cases, the uncorrelated lognormal (UCLN) relaxed-clock model was preferred. For the model-based demographic inference, the non-parametric *skygrid* model was preferred for DENV-1 and DENV-3, and the parametric Exponential growth model for DENV-2 and DENV-4 [S4 Table].

Viral demographic curves were reconstructed for each serotype using either the isolates from Colombia (n = 235) sampled between 1982 and 2015 or the isolates from the region of Santander (n = 160) collected between 1998 and 2015 in order to have a measure of dengue genetic diversity and its fluctuations over time.

The pattern of DENV spread was identified using a standard continuous-time Markov chain (CTMC) coupled with the Bayesian stochastic search variable selection (BSSVS) procedure [38]. We assumed that the transition rates between locations were reversible (symmetrical model). Different schemes of discrete geographical locations were used [S3 Fig] to account for any sampling bias: in the first scheme, the country of isolation was used. In the second scheme, neighboring countries were grouped in regions except for Colombia and Venezuela: Andes (Bolivia, Ecuador and Peru), Central America, Greater Antilles, Lesser Antilles, North America and Southern Cone (Argentina, Brazil and Paraguay). In the final scheme, the Andean region and Southern cone regions were merged into one region named South American region. A Bayes factor test (BF > 9) was used to recognize well-supported diffusion rates using the SpreaD3 v0.9.6 software [39]; well-supported diffusion routes concerning Colombia were compared among schemes.

Bayesian approach was implemented in BEAST v1.8.3 software package [40]. For every analysis, five independent Markov Chain Monte Carlo (MCMC) were used with at least 100 million generations each. Samples were combined and diagnosed using Tracer v1.6 (http://tree.bio.ed.ac.uk/software/tracer) until the reached effective sample sizes over 200 for all parameters [41]. Maximum clade credibility trees (MCC) were summarized using TreeAnotator v1.8.1.

## Results

### DENV genotypes circulating in Colombia

A total of 235 full-length E-gene sequences from Colombia viruses isolated in Colombia were used in this study: 193 from the region of Santander (160 from the metropolitan area of Bucaramanga and 33 from the Province of Ocaña); 31 sequences were from the states of Antioquia (n = 21), Valle del Cauca (n = 6), Bolivar (n = 2), Guaviare (n = 1), and Cesar (n = 1); and 11 sequences for which the exact state was not available.

The nucleotide diversity (π)—the average number of nucleotide differences per site in pairwise comparisons among sequences—for the Colombian strains was similar for DENV-2 and DENV-4 (0.020) and lower for DENV-3 (0.015), and DENV-1 (0.011). The haplotype diversity (h)—the probability that two randomly sampled alleles are different—was high and nearly similar for all serotypes (DENV-1, 0.98; DENV-2, 1.0; DENV-3, 0.99; DENV-4, 0.99).

### Evolutionary history of DENV in Colombia

We documented DENV-1 genotype V, DENV-2 genotype Asian/American, DENV-3 genotype III, and DENV-4 genotype II in Colombia [S2 Fig]. The estimated mean rates of nucleotide substitution per site and per year (expressed as the mean and the 95% highest posterior density interval, HPD) were 6.14 x 10^−4^ (5.07–7.11), 7.30 x 10^−4^ (6.11–8.58), 8.80 x 10^−4^ (6.53–9.66), and 7.48 x 10^−4^ (5.96–9.04) for DENV-1 to DENV-4, respectively. These estimates were in line with those previously reported for the same genotypes circulating in the Americas [42–46]. For every genotype, we evidenced multiple introductions into Colombia as detailed below.

### DENV-1

**Fig 2:**
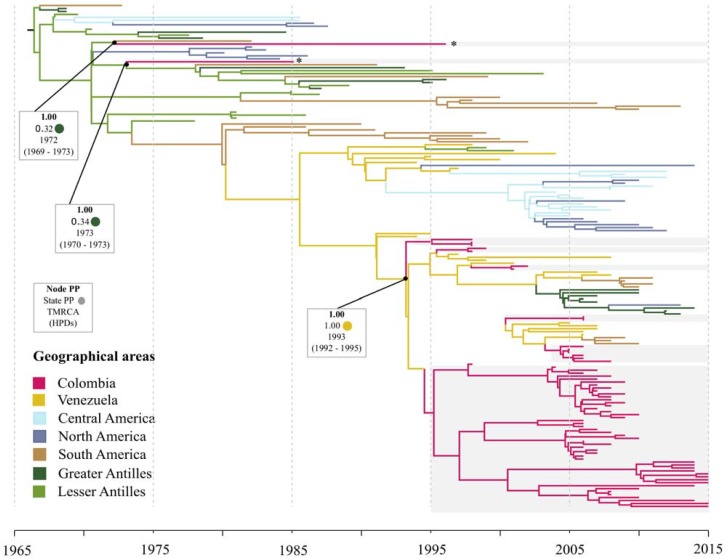
Maximum clade credibility tree of DENV-1. Branches colored according to the most probable posterior location of their child nodes. Monophyletic clades from Colombia highlighted in grey. Node posterior probability (bold), ancestral state probability and the mean estimated time to the most recent common ancestor (tMRCA) with the respective 95% highest posterior density interval (HPD) are indicated for relevant clades. *: virus strains isolated in 1985 (AF425616) and 1996 (AF425617) available in Genbank.

The MCC tree for DENV-1 included 79 E-gene sequences from Colombia and evidenced several introductions intro the country [Fig 2]. Two different virus strains isolated in 1985 (AF425616) and 1996 (AF425617) were related to viruses circulating in the Lesser Antilles and had an estimated time to the most recent common ancestor (TMRCA) around the early 1970s. Most of the strains isolated during the 1990s and the majority of the strains isolated during the 2000s (all of them sampled in the region of Santander) grouped in a different clade that intermixed with strains from Venezuela (1994–2008) and had a TMRCA around 1993 (95% HPD 1992–1995). This clade also included strains from other South America regions (Brazil/Paraguay/Argentina, 2008–2011), Greater Antilles (Dominica/Puerto Rico/Haiti, 2006–2014) and the United States of America (2013). Venezuela appeared as the most probable ancestral location (PP = 1.00) for the latest introductions of DENV-1 into Colombia.

### DENV-2

**Fig 3:**
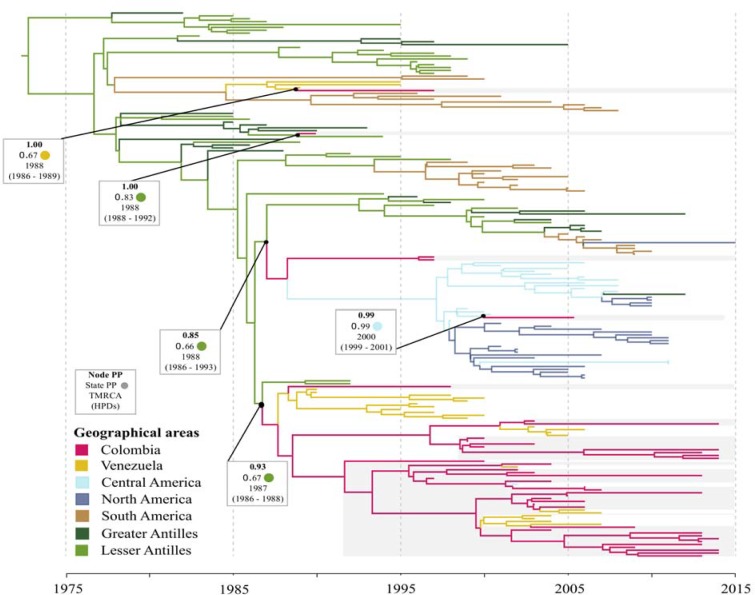
Maximum clade credibility tree of DENV-2. Branches colored according to the most probable posterior location of their child nodes. Monophyletic clades from Colombia highlighted in grey. Node posterior probability (bold), ancestral state probability and the mean estimated time to the most recent common ancestor (tMRCA) with the respective 95% highest posterior density interval (HPD) are indicated for relevant clades.

The majority of Colombian strains (38 out of 42) were from the Santander Region, and the remaining isolates were from the states of Antioquia (2004, n = 1), Guaviare (2005, n = 1) and Valle del Cauca (2015, n = 1). The MCC tree evidenced at least five different introductions of DENV-2 intro the country [Fig 3]. A single Colombian strain isolated in 1993 (location unknown) grouped with viruses from the Lesser Antilles (Trinidad and Tobago from 1997) and together they had a TMRCA around 1988 (95% HPD: 1988–1992). Another two strains isolated Santander (1998) grouped either with a few strains from Venezuela (1990–1996) or with strains from Central American (1999–2012) and, in each case, they had an estimated TMRCA around 1988 (95% HPD: 1986–1989 and 1986–1993, respectively). Viral strains sampled in the 1990s and thenceforth, grouped with the remaining strains from Venezuela (1991–2008) and with one strain from Costa Rica (2003); this group had a TMRCA around 1987 (95% HPD: 1986–1988) and was most likely introduced from the Lesser Antilles (PP = 0.93). Finally, a single strain isolated in 2007 from unknown locality did not fell in the previous group and instead clustered with viruses from Central America with a TMRCA around 2000 (95% HPD: 1999–2001); this case represents the most recent DENV-2 documented introduction into the country and possible a lineage that circulated in low-levels.

### DENV-3

**Fig 4:**
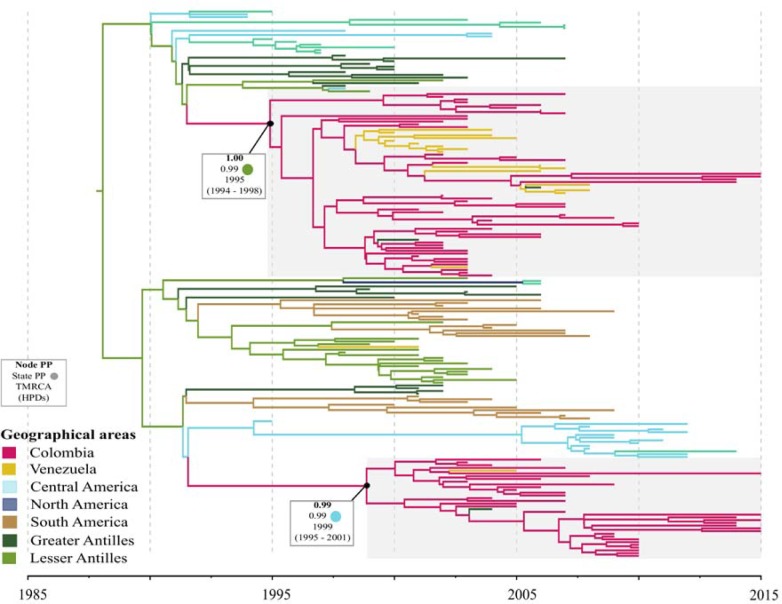
Maximum clade credibility tree of DENV-3. Branches colored according to the most probable posterior location of their child nodes. Monophyletic clades from Colombia highlighted in grey. Node posterior probability (bold), ancestral state probability and the mean estimated time to the most recent common ancestor (tMRCA) with the respective 95% highest posterior density interval (HPD) are indicated for relevant clades.

Our analysis included 89 E-gene sequences from Colombia and we identified two distinct lineages that co-circulated [Fig 4]. The first lineage included strains from Santander (2001–2015, n = 38), Norte of Santander (2005–2006, n = 5) Antioquia (2002–2007, n = 8), and one strain from Valle del Cauca (2014) that grouped together with strains from Venezuela (2000–2008) and the Greater Antilles (Puerto Rico, 2006; Cuba, 2001). This lineage was probably introduced from the Greater Antilles (PP = 0.81) and its TMRCA was around 1995 (95% HPD: 1994–1998). The second lineage consisted of the remaining 37 strains from multiple states from Colombia (Santander (2007–2015, n = 18), Antioquia (2003–2009, n = 12); Valle del Cauca (2004–2015, n = 3), Bolivar (2006 and 2015), and two strains for which the precise location was not available (2007, n = 2)) that grouped together with strains from Puerto Rico (2004) and Venezuela (2005). This second lineage was probably introduced from Central America (PP = 0.67) and its TMRCA was around 1999 (95% HPD: 1995–2001).

### DENV-4

**Fig 5:**
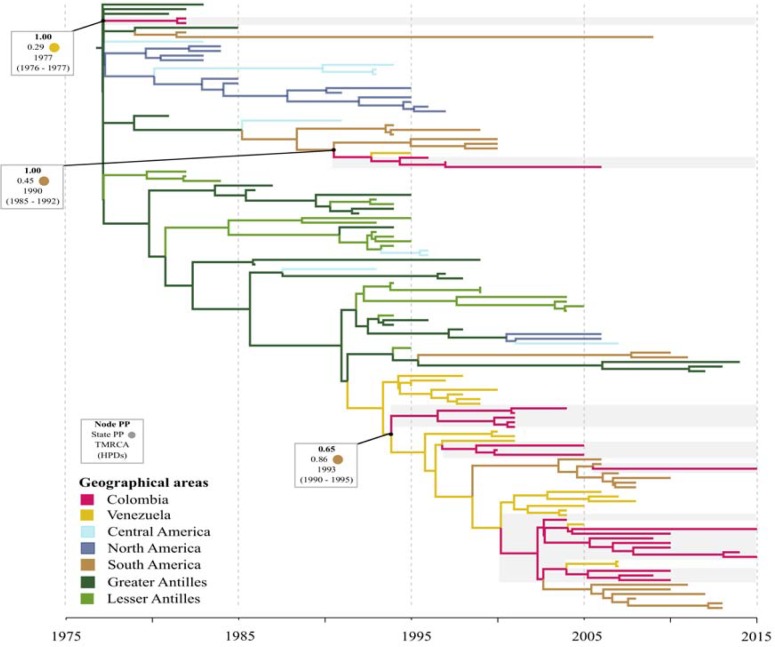
Maximum clade credibility tree of DENV-4. Branches colored according to the most probable posterior location of their child nodes. Monophyletic clades from Colombia highlighted in grey. Node posterior probability (bold), ancestral state probability and the mean estimated time to the most recent common ancestor (tMRCA) with the respective 95% highest posterior density interval (HPD) are indicated for relevant clades.

The MCC tree for DENV-4 included 25 E-gene sequences from Colombia and evidenced at least two introductions [Fig 5]. The majority (n = 19) of strains were from the Santander Region, two strains (2015) were from the states of Valle del Cauca and Cesar, and there were four strains for which the precise location was not available. The two oldest available isolates from Colombia (KC963424 and GU289913) sampled in 1982 grouped with strains from the Greater Antilles, North, South and Central America, and had a TMRCA around 1977 (95% BCI:1976–1977). Most Colombian strains sampled in 2000–2015 (20 out of 25) grouped with strains from Venezuela (1995–2008), and the majority of strains from other South American countries (Ecuador, Peru and Brazil, 2006–2013). The inferred TMRCA for this group was around 1993 (95% BCI: 1990–1995), and it was most likely introduced from Venezuela (PP = 0.65). The remaining three strains from Colombia, which were isolated in 1996, 1997 and 2006, grouped with one strain from Venezuela (1995), one from Ecuador (2000) and one from Peru (2000). This small group had a TMRCA around 1990 (95% HPD: 1985–1992). The strains from the region of Santander and the neighbor state of Cesar were closely related to strains from Venezuela and Brazil strains, while the single strain from Valle del Cauca (in the southwest part of Colombia) was closely related to strains from Ecuador and Peru.

### Well-supported migration rates concerning Colombia

We estimated the most significant migration routes among geographical locations using a discrete Bayesian phylogeographic analysis. To account for any sampling bias, the reconstructions were done using three different geographic schemes—detailed in methods. Well-supported diffusion routes with respect to Colombia were compared among schemes. In all cases, the recovered phylogeographical pattern underscored a strong link between Colombia and Venezuela in terms of viral diffusion. Likewise, the Caribbean region played an important role in the diffusion of DENV2—DENV4. Lastly, Central America was relevant for the diffusion of DENV2, whereas South America was relevant for the diffusion of DENV4.

**Fig 6:**
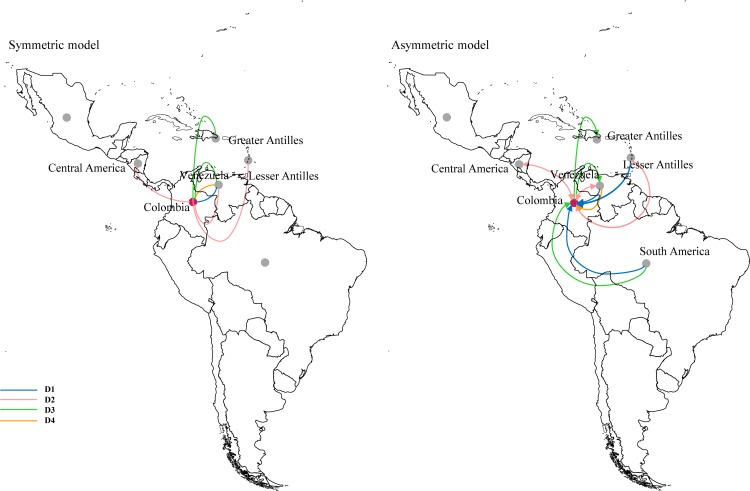
Summary of dengue virus (DENV) lineages spread with respect to Colombia. Colors indicate each serotype. Only routes with Bayes factor support > 9 are presented.

The most homogenous schema (scheme C) was also analyzed under an asymmetrical model as a proxy for directionality [Fig 6]. This analysis showed that most DENV lineages where introduced into Colombia from the other regions. Colombia appeared to be important for the diffusion of DENV3 into Venezuela and the Greater Antilles and had a significant bidirectional diffusion of DENV-2 lineages with Venezuela and Central America.

### DENV serotypes demographic history

**Fig 7:**
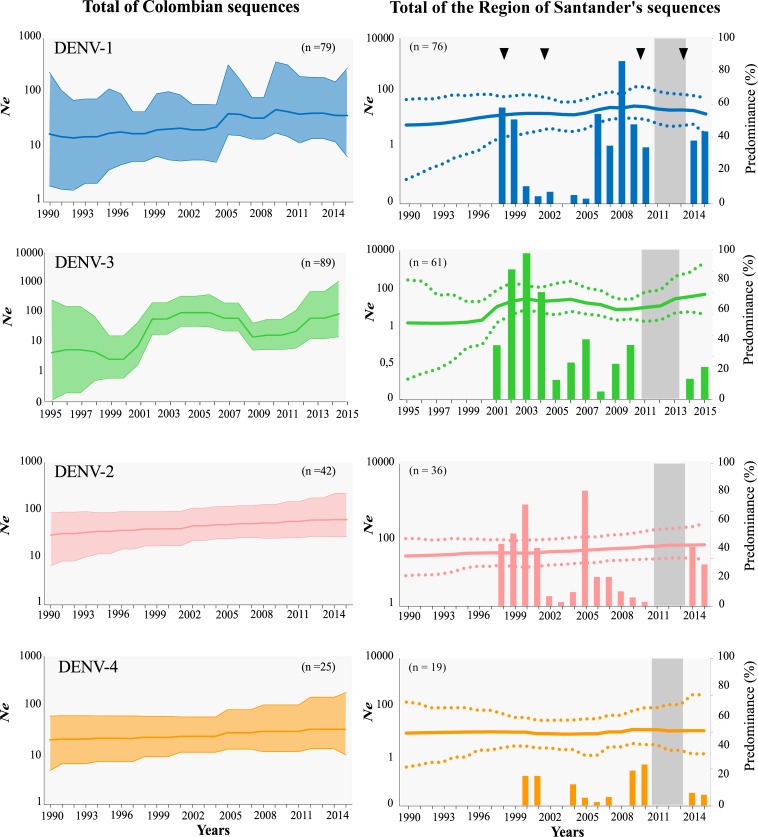
DENV Population dynamics in Colombia and the state of Santander (1998–2015). Left panels: relative genetic diversity of each serotype in Colombia estimated using non-parametric Skygrid coalescent analysis. Right panels: relative genetic diversity of each serotype in the state of Santander overlapped with the respective serotype predominance. National dengue epidemics indicated with triangles. Gray colored areas show the periods that were not sampled. *Ne* denotes effective population size in logarithmic scale.

The Bayesian demographic reconstructions for Colombia are shown in Fig 7. Overall, we observed a steady increase in the effective population size (*Ne*) over time for DENV-2 and DENV–4. There were oscillations in the case of DENV-1 and more pronounced ones in the case of DENV-3, but the general trend is of increased diversity over time. In the case of DENV-3, there is an increase in diversity peaking during the period 2001–2005, a sharp drop after that and a steady increase from 2009 onwards. The demographic reconstruction for the region of Santander, which accounted for 82% of the Colombian sequence data, followed the same dynamics as that recovered with the dataset from the Country.

There was an agreement between the increase of *Ne* of DENV-3 and the re-introduction of this serotype into the country in 2001 that led to high DENV-3 prevalence in the following years. The same type of pattern was not observed for DENV-1, which was highly prevalent during the periods of 1998–1999 and 2007–2008. DENV-2 and DENV-4 are respectively the most and less prevalent serotypes isolated in the region and although there have been changes in the prevalence of both of them over time, we did not observe any visible difference in the growth of *Ne*.

## Discussion

This study is the first large-scale analysis of the spatial and temporal dynamics of DENV in Colombia during the period 1998–2015 using newly sequenced and curated genetic data retrieved from GenBank. The novel sequences were sampled in distinct locations of Colombia, but most of them came from the region of Santander. We documented the frequent introduction of dengue lineages into Colombia, the ongoing co-circulation of multiple serotypes and lineages within the country and the local viral population dynamics.

Our phylogenetic analysis confirmed the circulation of one genotype per serotype (DENV-1: Genotype V, DENV-2: Asian/American Genotype, DENV-3: Genotype III and DENV-4: Genotype II) and it is consistent with the results of other studies [16–19]. The presence of DENV-4 genotype I was documented in Brazil in 2013 [47], but we did not find evidence of its circulation in Colombia. Despite multiple strain introductions of DENV-1, DENV-2 and DENV-4, our data indicated that only one lineage predominates up to date, suggesting the turnover of viral lineages over time. With the data at hand we could not address if such turnovers are due to stochastic die-off or other factors; however, the re-emergence of DENV-2 in an dengue-endemic setting from Indonesia was due to the loss or decrease of herd immunity during a 5-year period where DENV-1 predominated [48]. Similarly, changing serotype prevalence has been associated with lineage extinction and lineage replacements in Thailand and with dengue epidemics in Singapore [49, 50, 51, 52]. Here, we recorded two distinct lineages of DENV-3 that apparently were introduced at the same time, that had the same level of circulation and that overlapped in time and space. It may be the case that, the complete susceptibility of the population to this serotype at the time of the introduction allowed two successful independent transmission chains. Similarly, the co-circulation of two DENV-3 lineages has been reported in the metropolitan area of Medellin, Colombia [18] and the co-circulation of different lineages has been demonstrated for DENV-1 and DENV-2 in Brazil [53,54].

Our estimated tMRCAs for each serotype preceded (between 5–7 years) the respective official epidemiologic reports by the National Institute of Health from Colombia [9,12]. This is likely the outcome of a preliminary silent circulation of virus (*i*.*e*., individuals experiencing mild to asymptomatic infections) coupled with local passive surveillance for dengue [2]. The DENV surveillance in Colombia relies on the immediate reporting of fatal cases and the weekly routine reporting of symptomatic ones by the health care providers [55]. Consequently, the true burden of dengue might be underestimated once local physicians may fail to report cases quickly and routinely. Such passive surveillance includes neither a thorough serotyping nor the genetic characterization of strains owing to financial costs and time constraints. All fatal cases are laboratory-confirmed, but only around 10–20% of laboratory confirmations remain mandatory for the non-fatal ones [55]. Thus, health care providers belonging to the public health sentinel surveillance system send only a small fraction of collected serum samples to the National Institute of Health for laboratory confirmation and serotype identification. As a consequence, an epidemic has often reached or passed its peak before it has been recognized or before it can be controlled, resulting in a vague idea of the influence of the serotypes behind it.

The increased regional commerce and travel in dengue hyper-endemic Latin America likely drive frequent exchanges and importation of DENV through human movement. For instance, it has been shown an effect of air travel in increased dengue transmission in the Americas and Asia [43, 53, 56]. Here, We observed frequent viral lineage exchange among Latin American countries and persistent co-circulation of dengue viral lineages. Human flow in Colombia is also particularly significant as the country is the largest sending country of migrants in South America [57,58]. However, the country seems to act as a sink instead of a source for DENV lineages. The strong association with Venezuela probably reflects geographical proximity, connectivity through trade and migratory history (A significant migrant wave occurred in the mid 80’s to Venezuela, primarily motivated by its economic boom and by the economic difficulties of Colombia at that time). However, we also do not rule out the possibility that this association could be a result of intense sampling in the northeast of Colombia. For example, the DENV-4 sequence from Valle del Cauca (southwestern Colombia) was related to those from Peru and Ecuador. Nevertheless, the region of Santander represents a point of transit with the neighboring country of Venezuela and thus substantial morbidity due to cross-border exchange of dengue-infected people is expected to continue.

Given that DENV transmission is similar to other arboviruses, the aforementioned high levels of viral exchange are consistent with the rapid and recent establishment of chikungunya (CHIKV) and Zika (ZIKV) in the region [59,60,61]. However, whereas the dynamics of DENV are complicated by the four serotypes and complex susceptibility profiles of local populations, recurring CHIKV and ZIKV outbreaks will be functionally related to the turnover rate of susceptible humans in addition to reintroduction rates [62]. These observations underscore that arboviral diseases are a recurring public health problem in Colombia and recognizing the importance of their molecular investigation will strengthen diagnosis and epidemiological integrated surveillance.

The population dynamics of DENV can be driven by viral introduction events that could eventually lead to lineage turnovers as have been documented in other settings [43,53,63,64, 65]. For instance, regular stochastic clade replacement led to recurring homotypic dengue outbreaks in Malaysia [66]. In the same vein, constant DENV introductions and *in situ* evolution contribute to viral diversity in Singapore where replacement of a predominant viral clade, even in the absence of a switch in the predominant serotype, hinted a possible increase in transmission [52]. In our study, and in particular in the Santander Region, we observed the consistently co-circulation of the four serotypes over consecutive years. This underscores an intricate pattern of competition that does not result in complete serotype displacement and may explain the increase of diversity over time for the four serotypes. We observed much rapid rise in the *Ne* of DENV-3 followings its introduction that was also reflected in the predominance of this serotype in the period 2001–2004. Thus, in the Santander Region, both *in situ* evolution and the recurrent introduction of lineages drive local dengue dynamics.

This study had limitations: most of the samples were collected from a single region and this limits our knowledge on the country-wide viral genetic diversity. Likewise, samples from other countries and times are quite heterogeneous and this might also hide additional routes and the relevance of specific regions from the establishment and transmission of dengue in Colombia and the Americas. Furthermore, giving scarcity of resources, limited data on serotype prevalence restricts the conclusion that could be drawn on the dynamics of DENV. Larger series with long-term follow-up are needed to confirm the effect that a particular serotype might have during epidemics. Nonetheless, even with these limitations, we showed that passive laboratory-based disease surveillance studies allowed us to have a general picture of the diversity and dynamics of DENV.

## Conclusion

We characterized the spatial-temporal dynamics of all dengue serotypes in a highly endemic area of Colombia, documented the co-circulation of a single genotype of each serotype, and the unapparent circulation of every serotype prior its first detection in the country. This study also showed that genetic diversity of serotypes circulating in the country continues to grow due to *in-situ* evolution and recurring introductions of viral strains from different countries in the region. Our study advances the countrywide genomic surveillance to lay the groundwork for the introduction of dengue vaccines and future control initiatives and underscores the continued need for a sensitive surveillance system aiming to collect reliable baseline data.

## Supporting information

S1 Fig Phylogenetic signal analysis. Likelihood mapping diagrams were obtained using TREE-PUZZLE. All datasets had between 67% and 91,3% proportion of well resolved quarters. The index of substitution saturation calculated with DAMBE was below 1.0 (0,04–0,1) and smaller than the critical index of substitution saturation (0,4–0,8) indicating that the sequences did not experience severe substitution saturation.
(SVG)

S2 Fig Phylogeographical reconstruction. Three different schemes were analyzed under symmetrical models. The most homogenous schema (scheme C) was also analyzed under an asymmetrical model. Panels next to maps show the number of sequences included. BF denotes Bayes Factor.
(SVG)

S3 Fig Phylogenetic trees constructed with Maximum Likelihood methods. Trees were rooted using Yellow fever virus; every tree included representative taxa for each serotype and representative taxa for the corresponding genotype. Colored tips represent the samples from Colombia: DENV-1 Genotype V in blue, DENV-2 Genotype Asian/American in red, DENV-3 Genotype III in green, and DENV-4 Genotype II in yellow.
(SVG)

S1 Table Primers using to amplify and sequence DENV E-genes. (DOCX)

S2 Table DENV E gene sequences from Colombian viruses generated in this study. (DOCX)

S3 Table Clock-likeness using TempEst v 1. 5. (DOCX)

S4 Table Log marginal likelihood estimates for different coalescent model combinations. (DOCX)
