# Supplementary material for: Evolutionary history and spatio-temporal dynamics of dengue virus serotypes in an endemic region of Colombia
Source: PLoS One. 2018 Aug 29;13(8):e0203090. doi: 10.1371/journal.pone.0203090 (PMC6114916; doi:10.1371/journal.pone.0203090)
Supplement: S1 Table — (DOCX) [file pone.0203090.s004.docx]

| **Supplementary Table 1.** Primers using to amplify and sequence DENV E-genes. | | | |
| --- | --- | --- | --- |
| **Serotype / identifier** | **Genome position** | **Sequence** | **Base pairs** |
| DENV-1: |  |  |  |
| D1-Fwd1-E | 618 | 5´-CCAGATGACGTTGACTGCTGGTG-3´(S) | 1143 |
| D1-Rev1-E | 1704 | 5´-CGCAGTGTGCATTGCTCCTTC-3´ |  |
| D1-Fwd2-E | 1627 | 5´-GGAACAGACAAGATTTGCTGGT-3´(S) | 1155 |
| D1-Rev2-E | 2763 | 5´-GCTTTTCCCCAGCTTTTCCA-3´ |  |
| D1-MA-E | 1422 | 5´-GGAGCTTGAGGTGTTATGGT-3´(S) | 1017 |
| D1-MB-E | 2439 | 5´-AGTTCTCTGCCCTTCCAGTT-3´´ |  |
| DENV-2: |  |  |  |
| D2-Fwd1-E | 723 | 5’-TCTTGAGACATCCAGGCTTTAC-3´ (S) | 863 |
| D2-Rev1-E | 1586 | 5’-GAGACCCTAAGACAACGACATC -3´ |  |
| D2-Fwd2-E | 1476 | 5’- GGTTCCTAGACCTGCCATTAC -3´(S) | 851 |
| D2-Rev2-E | 2327 | 5’-TCACAACGCAACCACTATC -3´ |  |
| D2-MA-E | 990 | 5’-CCAAACAACCTGCCACTCTAA-3’ (S) | 1337 |
| D2-MB-E | 2420 | 5’-GGGATTCTGGTTGGAACTTGTA-3’ |  |
| DENV-3: |  |  |  |
| D3-Fwd1-E | 787 | 5'-GACCCAGAAGGCGGTTATTT -3´(S) | 1260 |
| D3-Rev1-E | 2026 | 5'-GCCTCGAACATCTTCCCAATA -3´ |  |
| D3-Fwd2-E | 1915 | 5'-GAAGGAGGAGCCTGTCAATAT -3´ (S) | 1175 |
| D3-Rev2-E | 3068 | 5'-TGTAGTTGTGTTGCGAGATAGG -3 |  |
| D3-MA-E | 951 | 5' - ACAAGCCCACGTTGGATATAG - 3' (S) | 1520 |
| D3-MB-E | 2471 | 5' - ATTCCGCACACTCCATTCTC- 3' |  |
| DENV-4: |  |  |  |
| D4-Fwd1-E | 854 | 5'-ATCCAGCGAACTGTCTTCTT -3´ (Se | 1078 |
| D4-Rev1-E | 1912 | 5'- GCTCCAGCACCTTCATACTT -3´) |  |
| D4-Fwd2-E | 1498 | 5'-CCCAGGTCTGGAATTGACTT -3´ (S) | 1300 |
| D4-Rev2-E | 2825 | 5'-AGGTGTCTGGTCCGTCTAT -3´ |  |
| D4-MA-E | 938 | 5'-GAATGCGATGCGTAGGAGTAG-3' (S) | 1438 |
| D4-MB-E | 2376 | 5'-AGTGATTCCTCCAACAGCTATG -3' ) |  |
| Fwd1, Rev1, Fwd2, Rev2: using for amplifying and sequencing; MA, MB: only for sequencing. | | | |
