# Supplementary material for: Evolutionary history and spatio-temporal dynamics of dengue virus serotypes in an endemic region of Colombia
Source: PLoS One. 2018 Aug 29;13(8):e0203090. doi: 10.1371/journal.pone.0203090 (PMC6114916; doi:10.1371/journal.pone.0203090)
Supplement: S2 Table — (DOCX) [file pone.0203090.s005.docx]

| **Supplementary Table 2**. DENV E gene sequences from Colombian viruses generated in this study. | | | | | | | | | |
| --- | --- | --- | --- | --- | --- | --- | --- | --- | --- |
| **Serotype** | | **Year** | **GenBank** | | **Serotype** | | **Year** | **GenBank** | |
| DENV-1 | | 2015 | MF817445 | | DENV-1 | | 2006 | JQ581639 | |
|  | | 2015 | KX901656 | |  | | 2006 | JQ581645 | |
|  | | 2015 | MF817446 | |  | | 2006 | JQ581606 | |
|  | | 2015 | MF173468 | |  | | 2006 | JQ581607 | |
|  | | 2015 | MF173467 | |  | | 2006 | JQ581608 | |
|  | | 2015 | MF173466 | |  | | 2006 | JQ581609 | |
|  | | 2014 | MF173465 | |  | | 2006 | JQ581610 | |
|  | | 2014 | MF173464 | |  | | 2006 | JQ581611 | |
|  | | 2014 | KX901655 | |  | | 2006 | JQ581612 | |
|  | | 2014 | MF173463 | |  | | 2006 | JQ581614 | |
|  | | 2014 | MF173462 | |  | | 2006 | JQ581615 | |
|  | | 2014 | MF173461 | |  | | 2006 | JQ581616 | |
|  | | 2010 | KX901654 | |  | | 2006 | JQ581621 | |
|  | | 2010 | MF173460 | |  | | 2006 | JQ581635 | |
|  | | 2010 | MF173459 | |  | | 2006 | JQ581640 | |
|  | | 2010 | MF173458 | |  | | 2006 | JQ581641 | |
|  | | 2009 | KX901653 | |  | | 2006 | JQ581642 | |
|  | | 2009 | MF173457 | |  | | 2006 | JQ581644 | |
|  | | 2009 | MF817447 | |  | | 2006 | JQ581646 | |
|  | | 2009 | MF173456 | |  | | 2006 | JQ581647 | |
|  | | 2009 | MF173455 | |  | | 2005 | JQ581613 | |
|  | | 2008 | JQ581604 | | DENV-2 | | 2015 | KY887608* | |
|  | | 2008 | JQ581619 | |  | | 2015 | KY887628 | |
|  | | 2008 | JQ581634 | |  | | 2015 | KX901652 | |
|  | | 2008 | JQ581602 | |  | | 2015 | KY887616 | |
|  | | 2008 | JQ581620 | |  | | 2015 | KY887614 | |
|  | | 2008 | JQ581624 | |  | | 2015 | KY887617 | |
|  | | 2008 | JQ581631 | |  | | 2015 | KY887627 | |
|  | | 2008 | JQ581630 | |  | | 2014 | KY887621 | |
|  | | 2008 | JQ581623 | |  | | 2014 | KX901651 | |
|  | | 2008 | JQ581603 | |  | | 2014 | KY887618 | |
|  | | 2008 | JQ581636 | |  | | 2014 | KY887613 | |
|  | | 2007 | JQ581627 | |  | | 2014 | KX901650 | |
|  | | 2007 | JQ581629 | |  | | 2014 | KY887619 | |
|  | | 2007 | JQ581633 | |  | | 2014 | KY887629 | |
|  | | 2007 | JQ581637 | |  | | 2014 | KY887615 | |
|  | | 2007 | JQ581622 | |  | | 2010 | KY887609 | |
|  | | 2006 | JQ581638 | |  | | 2010 | KX901649 | |
|  | | 2006 | JQ581643 | |  | | 2008 | KY887611 | |
| **Supplementary Table 2**. Continue. | | | | | | | | | |
| **Serotype** | **Year** | | | **GenBank** | **Serotype** | **Year** | | | **GenBank** |
| DENV-2 | 2007 | | | KY887622 | DENV-3 | 2010 | | | KX926480 |
|  | 2007 | | | KY887620 |  | 2009 | | | KX926479 |
|  | 2006 | | | KY887623 |  | 2009 | | | KX926478 |
|  | 2005 | | | KY887612 |  | 2009 | | | KX926477 |
|  | 2005 | | | KY887607 |  | 2008 | | | KX926476 |
|  | 2004 | | | KY887610 |  | 2007 | | | KX926474 |
|  | 2004 | | | KY887624 |  | 2007 | | | KX926475 |
|  | 2003 | | | KX901648 |  | 2007 | | | KX926473 |
|  | 2001 | | | KX901647 |  | 2003 | | | KX926472 |
|  | 2001 | | | KY887625 |  | 2003 | | | KX926471 |
|  | 2001 | | | KY887626 |  | 2003 | | | KX926470 |
|  | 2000 | | | KX901646 |  | 2003 | | | KX926469 |
|  | 1998 | | | KX901645 |  | 2003 | | | KX926468 |
|  | 1998 | | | KY887630 |  | 2003 | | | KX926467 |
|  | 1998 | | | KY887631 |  | 2002 | | | KX926466 |
| DENV-3 | 2015 | | | MF173451* |  | 2002 | | | KX926465 |
|  | 2015 | | | KX910787 |  | 2002 | | | KX926464 |
|  | 2015 | | | MF173452 | DENV-4 | 2015 | | | KX901661 |
|  | 2015 | | | MG009511* |  | 2015 | | | KY887637* |
|  | 2015 | | | MF173489 |  | 2015 | | | KY887636* |
|  | 2015 | | | MG009512 |  | 2014 | | | KX901660 |
|  | 2015 | | | MG009513 |  | 2010 | | | KY887635 |
|  | 2014 | | | MG009514 |  | 2010 | | | KX901659 |
|  | 2014 | | | MF173453* |  | 2010 | | | KX901658 |
|  | 2014 | | | MG009515 |  | 2010 | | | KY887634 |
|  | 2014 | | | MF173454 |  | 2010 | | | KY887633 |
|  | 2014 | | | KX910786 |  | 2009 | | | KY887632 |
|  | 2010 | | | KX926490 |  | 2009 | | | KX901657 |
|  | 2010 | | | KX926489 |  |  | | |  |
|  | 2010 | | | KX926488 |  |  | | |  |
|  | 2010 | | | KX926487 |  |  | | |  |
|  | 2010 | | | KX926486 |  |  | | |  |
|  | 2010 | | | KX926485 |  |  | | |  |
|  | 2010 | | | KX926484 |  |  | | |  |
|  | 2010 | | | KX926483 |  |  | | |  |
|  | 2010 | | | KX926482 |  |  | | |  |
|  | 2010 | | | KX926481 |  |  | | |  |
| Viruses from the Santanderes Region; *Viruses from Valle del Cauca, Cesar and Bolivar states. | | | | | | | | | |
