# Supplementary material for: Evolutionary history and spatio-temporal dynamics of dengue virus serotypes in an endemic region of Colombia
Source: PLoS One. 2018 Aug 29;13(8):e0203090. doi: 10.1371/journal.pone.0203090 (PMC6114916; doi:10.1371/journal.pone.0203090)
Supplement: S3 Table — (DOCX) [file pone.0203090.s006.docx]

| **Supplementary Table 3.** Clock-likeness using TempEst v 1. 5 | | | |
| --- | --- | --- | --- |
| **Serotype** | **Data of evolutionary reconstruction**  (American sequences) | **Data of Colombian demographic reconstruction** | |
|  |  | All Colombia seq | Santander seq |
| **DENV-1** | 0,83 | 0,476 | 0,61 |
| **DENV-2** | 0,77 | 0,534 | 0,63 |
| **DENV-3** | 0,6 | 0,659 | 0,86 |
| **DENV-4** | 0,85 | 0,84 | 0,69 |
| Values correspond to R2 of regression of root-to-tip genetic distance against sampling time. Seq: sequences. | | | |
