# Supplementary material for: Evolutionary history and spatio-temporal dynamics of dengue virus serotypes in an endemic region of Colombia
Source: PLoS One. 2018 Aug 29;13(8):e0203090. doi: 10.1371/journal.pone.0203090 (PMC6114916; doi:10.1371/journal.pone.0203090)
Supplement: S4 Table — (DOCX) [file pone.0203090.s007.docx]

| **Supplementary Table 4.** Log marginal likelihood estimates for different coalescent model combinations. | | | | | |
| --- | --- | --- | --- | --- | --- |
| **Serotype** | **Model** | **Evolutionary history** | | **Demographic history** | |
|  |  | **PS** | **SS** | **PS** | **SS** |
| **DENV-1** | Constant size | -8347 | -8348,25 | -3633,38 | -3633,45 |
|  | Exponential growth | -8347,64 | -8350,18 | -3624,41 | **-3624,35** |
|  | Skygrid | **-8328,47** | **-8328,95** | **-3624,16** | -3624,51 |
|  | Skyride | -8352,09 | -8354,7 | -3632,07 | -3632,49 |
| **DENV-2** | Constant size | -9384,26 | -9388 | -3719,14 | -3719,25 |
|  | Exponential growth | -9345 | -9349,3 | **-3714,21** | **-3714,45** |
|  | Skygrid | **-9344,53** | **-9346,21** | -3716,73 | -3716,96 |
|  | Skyride | -9363,59 | -9366,83 | -3716,07 | -3716,08 |
| **DENV-3** | Constant size | -8600,93 | -8603,07 | -4785,08 | -4785,05 |
|  | Exponential growth | -8565,61 | -8570,15 | -4783,05 | -4783,68 |
|  | skygrid | **-8556,34** | **-8558,89** | **-4766,52** | **-4767,41** |
|  | Skyride | -8576,3 | -8579,6 | -4776,1 | -4776,45 |
| **DENV-4** | Constant size | -7233,45 | -7233,99 | -3271,11 | -3271,24 |
|  | Exponential growth | -7222,99 | -7223,81 | **-3269,18** | **-3269,23** |
|  | Skygrid | -7209,7 | -7209,93 | -3269,76 | -3269,91 |
|  | Skyride | **-7175,99** | **-7105,21** | -3269,27 | -3269,42 |
| Evolutionary history data: DENV-1 (genotype V), DENV-2 (Asian- American genotype), DENV-3 (genotype III) and DENV-4 (genotype II) from America countries and Demographic history data: DENV-1, DENV-2, DENV-3 and DENV-4 from Colombia. The best fitting model is bold. Coalescent models used were the parametric Constant and Exponential models and the non-parametric Bayesian skyride and skyride model. PS = path sampling model selection, SS = stepping-stone model selection. | | | | | |
